# Flocculation and Expression of *FLO* Genes of a *Saccharomyces cerevisiae* Mezcal Strain with High Stress Tolerance

**DOI:** 10.17113/ftb.57.04.19.6063

**Published:** 2019-12

**Authors:** Israel Vergara-Álvarez, Francisco Quiroz-Figueroa, María Concepción Tamayo-Ordóñez, Amanda Alejandra Oliva-Hernández, Claudia Patricia Larralde-Corona, José Alberto Narváez-Zapata

**Affiliations:** 1 National Polytechnic Institute (Instituto Politécnico Nacional), Center for Genomic Biotechnology, Blvd del Maestro s/n esq, Elías Piña Col. Narciso Mendoza, C.P. 88710, Reynosa (Tamaulipas), Mexico; 2 Aix-Marseille University, LCB (UMR7283), CNRS, Marseille, France; 3 National Polytechnic Institute (Instituto Politécnico Nacional), CIIDIR-IPN Unidad Sinaloa, Blvd. Juan de Dios Bátiz Paredes no. 250, Col. San Joachin, C.P. 81101 Guasave (Sinaloa), Mexico; 4 Genetic Engineering Laboratory, Department of Biotechnology, Faculty of Chemical Sciences, Autonomous University of Coahuila, Saltillo Unit, Mexico

**Keywords:** *Saccharomyces cerevisiae*, flocculation, stress tolerance, fermentation, mezcal, agave must

## Abstract

Mezcal is a distillate produced by spontaneous fermentation of the must obtained from stalks of *Agave* spp. plants that are cooked and pressed. Agave must contains a high amount of fructose and phenolic compounds, and fermentation usually occurs under stressful (and uncontrolled) environmental conditions. Yeasts capable of growing under such conditions usually display advantageous biological and industrial traits for stress tolerance such as flocculation. In this study, seven *Saccharomyces cerevisiae* strains isolated from mezcal must were exposed to temperatures ranging between 10 and 40 °C, and to different sugar sources (fructose or glucose). Yeasts grown in fructose increased their stress tolerance, determined by colony count in a microdrop assay, under low temperature (10 °C) compared to the growth at 40 °C on solid cultures. The most stress-tolerant mezcal strain (Sc3Y8) and a commercial wine (Fermichamp) strain, used as control, were grown under fermentation conditions and exposed to long-term temperature stress to determine their performance and their potential for flocculation. Compared to glucose, fermentation on fructose increased the metabolite accumulation at the end of culture, particularly at 40 °C, with 2.3, 1.3 and 3.4 times more glycerol (8.6 g/L), ethanol (43.6 g/L) and acetic acid (7.3 g/L), respectively. Using confocal microscopy analysis, we detected morphological changes such as aggregation and wall recognition at the level of budding scars in yeast, particularly in the Sc3Y8 strain when it was exposed to 40 °C. The analysis confirmed that this mezcal strain was positive for flocculation in the presence of Ca^2+^ ions. Analysis of *FLO1*, *FLO5* and *FLO11* gene expression implicated in flocculation in both *Saccharomyces* strains showed a strong transcriptional induction, mainly of the *FLO5* gene in the mezcal Sc3Y8 strain.

## INTRODUCTION

Mezcal production process presents fluctuating environmental conditions (*i.e.* osmotic pressure, ethanol concentration, pH and temperature conditions) which might be stressful for yeasts (*1*, *2*). Particularly, mezcal must has a very different sugar composition from wine must. It is characterized by having a high concentration of fructose and low of glucose (up to 90 and approx. 10 g/L, respectively) (*3*, *4*). Yeasts that participate in the fermentation process might display special features to survive these stressful conditions (*5*, *6*) and according to their origin may be at different domestication stages (evolution events) (*7*). Phenotypic characterization has been widely documented in *Saccharomyces* wine strains (*8*–*10*), but only a few studies have reported these phenotypic traits in yeasts originating from mezcal must (*4*, *11*). This is important since mezcal yeasts reported here are part of a different domestication event as compared to other well reported *S. cerevisiae* strains, and therefore their phenotypic characteristics may also vary (*7*). This has been corroborated by characterization of their osmotic and ethanol tolerance, and it has been observed that it depended on the type of hexose present in the medium, specifically fructose (*11*). Fructose consumption is also affected by temperature in *Saccharomyces* strains (*12*). Although, to our knowledge, there are no studies focused on exploring the effect of this stressor on *Saccharomyce*s strains isolated from mezcal.

It has been proposed that some stress-tolerant yeasts may flocculate (*13*) and exhibit good growth and survival in suboptimal environmental conditions (*14*, *15*). Flocculation is a natural trait in *S. cerevisiae* and has been documented in brewer’s and wine strains (*16*, *17*). This asexual aggregation process is mediated by lectins (*18*) and is strain-dependent, or it may be triggered by different factors as nutrient depletion or alcohol accumulation (*19*–*21*). Temperature also affects the flocculation by acting at the level of cellular interactions (*19*). At molecular level, genes involved in this cellular aggregation have been identified, mainly those called *FLO* genes, which are classified according to their response to different environmental factors such as nutrients, pH, ethanol and temperature changes (*1*, *22*, *23*). In general, *FLO1*, *FLO5* and *FLO11* genes have been related to flocculation and cell aggregation in *Saccharomyces* strains (*10*, *24*). Specifically in wine strains, *FLO5* gene has recently been associated with the flocculation (*25*) and with cell wall organization and assembly (*26*).

Stress tolerance of yeasts, mainly under high fructose concentrations, is an important industrial trait since one of the primary problems of the alcoholic fermentation is the occurrence of stuck fermentation when glucose is depleted and the only fermentable sugar left is fructose (*6*). Therefore, the aim of this study is to add to the knowledge of the mechanisms involved in stress tolerance of yeasts adapted to high fructose concentrations. In order to do that, two physiologically related traits, flocculation and temperature stress tolerance, were characterized. For this reason, we analyzed a collection of *S. cerevisiae* strains isolated from mezcal must (*4*) looking for those yeasts tolerant to temperature stress (thermotolerant). Fermentative performance and potential of flocculation were determined in the strain that was the most tolerant to temperature. In addition, expression behaviour of important flocculation genes (*FLO1*, *FLO5* and *FLO11*) under optimal and suboptimal temperature conditions was analyzed.

## MATERIALS AND METHODS

### Yeast strains and culture conditions

Six yeast strains belonging to our laboratory culture collection (Center for Genomic Biotechnology, National Polytechnic Institute, Reynosa, Mexico), obtained from fermenting mezcal must, were cultivated in yeast extract peptone dextrose (YPD) agar (containing in g/L: yeast extract 10, casein peptone 20, agar 20 and glucose 20; Sigma-Aldrich, Merck, Monterrey, Mexico) (*4*). Yeasts were labelled as: LCBG-Sc3Y4, LCBG-Sc3Y3, LCBG-Sc3Y2, LCBG-Sc3D5, LCBG-Sc3Y5, LCBG-Sc3Y8, and ScFerm (commercial wine strain *S. cerevisiae* Fermichamp, DSM Food Specialties B.V., Amsterdam, The Netherlands) that was used as control. Before each assay, a loop of each strain was pre-cultured in 250-mL Erlenmeyer flask with 50 mL of YPD broth (Sigma-Aldrich, Merck) for 18 h at 30 °C and 200 rpm in an incubator (Environ Shaker 3527; Lab-Line Instruments, Inc., Melrose Park, IL, USA).

### Selection of stress tolerant yeasts

Two culture media were used on yeast extract peptone (YP) plates (in g/L: yeast extract 10, casein peptone 20 and agar 20; Sigma-Aldrich, Merck) with 20 g/L of either glucose (YPD) or fructose (YPF; both Sigma-Aldrich, Merck), to evaluate the influence of the carbon source on the stress tolerance of these yeasts. Selection of thermotolerant yeasts was conducted using a microdrop assay (*11*). The plates were incubated at 10, 15, 20, 30 and 40 °C, and colony forming units were counted every 24 h until there was a visible growth, varying from 24 up to 216 h at 10 °C. The strains that were able to grow in the whole temperature range in both hexoses were considered to be thermotolerant.

### Fermentation conditions

Stress-tolerant yeasts were cultured in YP broth (in g/L: yeast extract 10 and casein peptone 20; Sigma-Aldrich, Merck) supplemented with 100 g/L of either glucose (YPD) or fructose (YPF; both Sigma-Aldrich, Merck). An inoculum concentration of 1.5‧10^6^ cell/mL was used for all the experiments, which were carried out in 50-mL Falcon tubes (Corning Inc., Corning, NY, USA) capped with cotton stoppers and maintained for 60 h without agitation at 30 or 40 °C. Cell population was determined by Neubauer chamber counting at different time intervals. Experiments were performed in triplicate.

### Metabolite quantification

Residual reducing sugars in YPD or YPF broth were detected colorimetrically by a modified dinitrosalicylic acid (DNS) method (*27*) measuring the absorbance at 580 nm in a DU 650 spectrophotometer (Beckman Coulter, Brea, CA, USA). Calibration curves were prepared using glucose or fructose as standard (Sigma-Aldrich, Merck). Production of glycerol, acetic acid and ethanol was determined by HPLC analysis at different time intervals using an HO1200 HPLC equipment (Agilent Technologies, La Jolla, CA, USA) with an Aminex HPX-87 column with a flow of 0.5 mL/min and a mobile phase of H_2_SO_4_ (5 mM) in Milli-Q water (Millipore, Burlington, MA, USA) for 30 min, and coupled to a refractive index (RI) detector. The samples were centrifuged at 14 400x*g* for 5 min at 10 °C in a Spectrafuge 24D (Labnet International Inc., Edison, NJ, USA), then diluted (1:10, *V/V*) in the mobile phase and filtered through nylon membranes (pore size 0.45 μm, 47 mm, GVS Magna™; Thermo Scientific, Mexico City, Mexico). Calibration curves within ranges of 0-20 (ethanol), 0-10 (glycerol) and 0-10 g/L (acetic acid) were set up for commercial standards (Sigma-Aldrich, Merck). Finally, experiments were performed in triplicate.

### Construction of standard cell curve

Cell number was determined spectrophotometrically using a standard curve constructed with correlation of the absorbance measured at 600 nm (DU 650 spectrophotometer; Beckman Coulter) and the number of cells of each strain present in a Neubauer chamber. Each experiment was performed in triplicate. Linear regressions and their fitting were: y=2‧10^7^x, with R^2^=0.9961, and 2‧10^7^x with R^2^=0.954 for ScFerm and Sc3Y8, respectively.

### Flocculation analysis

Flocculation was determined using the microflocculation method (*21*). Total cell number was calculated by extrapolation of the absorbance values obtained in each standard cell curve, and the number of flocculent cells was calculated by subtracting the number of cells that remained in the suspension after stopping the agitation, according to Ogata (*28*). As a negative flocculent control, the assay was conducted using deionized water instead of EDTA solution (Sigma-Aldrich, Merck). Experiments were performed in triplicate.

### Confocal microscopy

Flocculation was analyzed by confocal microscopy. Briefly, selected flocculent and non-flocculent strains were grown at 30 and 40 °C as described above. ScFerm was selected as the non-flocculent yeast. Cells were resuspended in citrate buffer (50 mM, pH=4.0; containing 8 mM CaCl_2_; Sigma-Aldrich, Merck) and then 0.4% (*m*/*V*) Solophenyl Flavine 7GFE 500 (AK Scientific, Inc., Union City, CA, USA) was added. Incubation was conducted at room temperature in darkness. The samples were washed with citrate buffer. Images were visualized and acquired with a confocal microscopy TCS SP5 X (Leica Microsystems, New York, NY, USA) at magnifications 20× and 40× with and without 4.0 digital zoom (confocal microscopy software LAS X v. 2.0.2.14392; Leica Microsystems) at 490 and 510-550 nm for excitation and emission, respectively. Finally, deionized water instead of the citrate buffer served as a negative flocculent control.

### FLO1, FLO5 and FLO11 primer design and validation

Genomic DNA isolation was conducted in *S*. *cerevisiae* (Sc3Y8 and ScFerm) cells grown in YPD broth using the DNAzol kit (Molecular Research Center, Cincinnati, OH, USA). DNA amount and quality were determined by using NANODROP 2000 equipment (Thermo Scientific). DNA samples were adjusted at final concentration of 100 ng/μL. For real-time polymerase chain reaction (PCR) amplification of *FLO1* and *FLO11* genes, the following primer sets were used: FLO1-F 5´-ATGCCTCATCGCTATATGTTTTTG-3´ and FLO1-R 5´-GCTCCTGAGGCCACACTAGTTAG-3, and FLO11-F 5´-CCTCCGAAGGAACTAGCTGTAATT-3´ and FLO11-R 5´- AGTCACATCCAAAGTATACTGCATGAT-3´, which produce fragments of 68 and 103 bp, respectively (*23*). To amplify the *FLO5* gene, a primer set was preliminarily designed for this study (FLO5-F 5’-ATGACAATTGCACACCACTGC-3’ and FLO5-R 5’-ATATATGCTGCATTCGAATATGTGG-3’) from *S. cerevisiae* S288c (NM_001179342.1) sequence. Then, using this *FLO5* sequence, a new primer set was designed (FLO5-F2 5’-GCATCAGGAAGTACGGAAGTCA-3’ and FLO5-R2 5’-TGCTGCATTCGAATATGTGGA-3’), which corresponds to a 110-bp fragment. MEGA v. 4.1 (*29*) program was used to align and design these primer sets. PCR products were amplified in reaction mix that contained 2 µL MgCl_2_ (50 mM; Bioline, Taunton, MA, USA), 2 µL dNTP mix (100 mM; Bioline), 2.5 µL PCR buffer (Bioline), 1.5 µL each primer (50 ng), 0.2 U Taq polymerase (Bioline), and 3 µL DNA template (100 ng). Initial denaturalization time was 5 min at 94 °C, followed by 35 cycles of 1 min at 94 °C, 1 min at 61 °C and 1 min at 72 °C, with a final extension cycle of 10 min at 72 °C. PCR products were verified and purified from an agarose gel (1%) using Wizard SV gel and PCR clean-up kit (Promega, Madison, WI, USA). These PCR products were then bound and cloned in a pGEM-T vector (Promega) and transformed using *Escherichia coli* DH10B competent cells (Invitrogen, Carlsbad, CA, USA). *FLO1*, *FLO5* and *FLO11* gene identity was confirmed by sequencing with an ABI 377 DNA sequencer equipment (Applied Biosystems, Foster City, CA, USA) with universal M13 primers and the BigDye terminator v. 3.1 cycle sequencing kit (Applied Biosystems).

### Gene expression analysis

Expression of the *FLO1*, *FLO5* and *FLO11* genes was determined on selected (Sc3Y8 and ScFerm) strains grown on YPD broth at 30 and 40 °C. Cells were collected after 12, 24, 48, 60 and 72 h. RNA isolation was carried out using the TRI reagent kit (Molecular Research Center) according to manufacturer’s instructions. RNA (1 µg) was treated with 1 U RQ1 RNase-Free DNase I (Promega), adjusting the volume to 10 µL, and incubated for 30 min at 37 °C. Reaction was stopped by adding 1 µL RQ1 DNase Stop Solution (Promega) followed by an incubation for 10 min at 65 °C. Genomic DNA contamination was verified by using agarose gels at 1.3% and by PCR reaction as follows: RNA treated with DNase was used as negative amplification template for the *UBC6* gene. This encodes a constitutive ligase EI protein involved in the ubiquitin-protein degradation (*30*). Genomic DNA was used as positive amplification control with the primers (UBC6-F 5’-GATACTTGGAATCCTGGCTGGTCTGTCTC-3’ and UBC6-R 5’-AAAGGGTCTTCTGTTTCATCACCTGTATTTGC-3’). PCR products were amplified in a 25-µL reaction containing 2 µL MgCl_2_ (50 mM; Bioline), 2 µL dNTP mix (100 mM; Bioline), 2.5 µL PCR buffer (Bioline), 1.5 µL each primer (50 ng), 0.3 µL Taq polymerase (Bioline), and 3 µL genomic DNA template (100 ng) or RNA treated with DNase (50 ng). Initial denaturalization time was 5 min at 94 °C, followed by 35 cycles at 94 °C for 1 min, 1 min at 59 °C and 1 min at 72 °C, with a final extension cycle at 72 °C for 10 min. cDNA synthesis was carried out using 1 µg RNA and the GoScript^TM^ Reverse Transcription System (Promega). Briefly, a final reaction volume of 20 µL containing 1 μg of DNase I‐treated RNA was mixed with 0.5 μg Oligo(dT)15 and random primers. Then, the reaction was incubated at 70 °C for 5 min, chilled in ice, and an RT Master Mix containing GoScript™ 1× reaction buffer, 5 mM MgCl_2_, 0.5 mM each dNTP, 1 U Recombinant RNasin Ribonuclease Inhibitor and 15 U/μg GoScript™ Reverse Transcriptase was added. Reaction was incubated at 25 °C for 5 min, then at 37 °C for 1 h, and finally at 70 °C for 15 min. A kanamycin-positive control of RNA provided in the kit was used to estimate the yield of cDNA synthesis. cDNA amount was verified in a NANODROP equipment (NANODROP-1000; Thermo Scientific) and kept at -60 °C until its use.

Expression of *FLO* genes was determined by the ΔΔCq method (*31*) using *UBC6* gene as a reference (*30*). Amplification was standardized in a real-time thermal cycler (GeneAmp PCR-System 9700; Applied Biosystems). Briefly, 50 ng/µL cDNA were used as template in 20 μL PCR reaction mix containing 10 μL PCR Master Mix (2X; Bioline) with SYBR Green (Applied Biosystems), and 0.5 pmol of each forward and reverse primer set. Conditions of amplification were similar to the previous section. In addition, negative control for each gene was included. Fluorescent signal of SYBR Green was standardized using the ROX dye included in the SYBR Green PCR Master Mix (Applied Biosystems).

### Data analysis

Gene relative expression (R) was calculated using the following equation:

R=(E_Ref_)C_tRef_/(E_Target_)C_tTarget_ /1/

where E_Ref_ and E_Target_ are the efficiencies of the reference and target gene, respectively, and C_tRef_ and C_tTarget_ are the threshold cycle (C_t_) average values of the reference and target gene, respectively (*31*). Significant differences (p<0.05) among the mean values were determined by Tukey’s test using one-way ANOVA and StatSoft Statistica software, v. 8.0 (*32*).

## RESULTS AND DISCUSSION

### Selection of thermotolerant strain

The analysis of yeast growth at different temperatures (10, 15, 20, 30 or 40 °C) showed that the carbon source affected the growth behaviour since all the *Saccharomyces* strains grew on fructose at all the temperatures (Table 1), but only Sc3Y8 strain was able to grow on glucose at 10 °C. De la Torre-González *et al*. (*11*) reported that this Sc3Y8 strain was able to grow at concentrations of fructose up to 850 g/L at 29 °C, and in an ethanol shock test, it was able to survive at 25% ethanol (*4*). Previously, it had been documented that lower temperatures affect the fructose consumption in *Saccharomyces* strains (*12*) and that this sugar may also decrease the osmotic pressure and ethanol stress tolerance level in *Saccharomyces* and non-*Saccharomyces* strains (*11*). The differences in stress tolerance that these strains show are in concordance with the theory of stress cross-response (*15*). Finally, the Sc3Y8 strain was selected for further studies because of its thermotolerance in the two sugar sources analyzed.

**Table 1:** Colony counts obtained by using the microdrop assay in yeast extract peptone dextrose (YPD) or yeast extract fructose (YPF) plates at different temperatures

*Saccharomyces* strain	*N*(colony)/(CFU∙10^6^/mL)																	
YPD plate										YPF plate							
Temperature/°C																	
10		15		20		30	40			10	15		20	30		40	
ScFerm	0	1.3±0.2		1.5±0.4		2.1±0.8		1.5±0.2			1.5±0.4	1.2±0.1	1.8±0.1		2.3±0.3	1.3±0.4	
Sc3Y8	2.6±0.3	2.5±0.3		4.8±0.3		3.9±1.3		2.3±0.2			3.8±0.5	2.4±0.5	4.6±0.1		4.5±0.3	2.5±0.5	
Sc3Y5	0	1.9±0.2		2.2±0.4		2.0±0.6		2.4±0.3			3.3±0.4	1.9±0.2	3.2±0.3		4.0±1.0	2.2±0.6	
Sc3D5	0	2.7±0.3		3.3±0.2		2.9±0.1		3.4±0.4			2.7±1.1	3.0±0.4	2.9±0.9		2.4±0.2	2.8±0.6	
Sc3Y2	0	6.5±0.4		7.0±1.4		11.4±0.7		8.1±1.6			7.5±0.8	6.0±0.9	6.7±1.1		11.4±0.6	7.4±0.3	
Sc3Y3	0	1.8±0.5		3.1±0.5		2.5±0.3		2.0±0.5			3.8±1.2	2.0±0.2	3.3±0.3		3.2±0.2	2.0±0.4	
Sc3Y4	0	1.8±0.3		2.4±0.3		3.4±0.7		2.5±0.7			2.9±0.6	2.7±0.8	2.6±0.5		4.0±0.2	1.7±0.1	

### Fermentation profiles

**Fig. 1:**
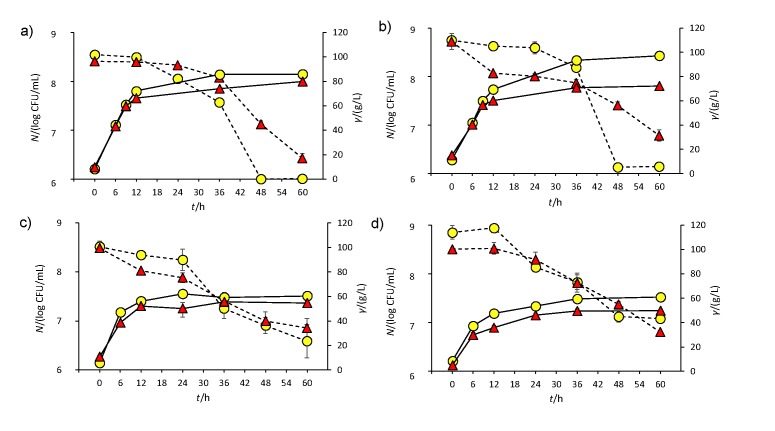
Cell counts and sugar consumption of *Saccharomyces cerevisiae* Sc3Y8 (red triangles) and ScFerm (yellow circles) strains grown in yeast extract peptone dextrose and fructose (YPD and YPF) broths at 30 (a and b, respectively) and 40 °C (c and d, respectively). Solid and dotted lines indicate the sugar consumption and cell count, respectively

The flocculation capability of the most thermotolerant mezcal strain, Sc3Y8, was further analyzed and compared to an industrial wine strain (ScFem), which is known to ferment grape must sugars to dryness (*33*). Both selected strains were analyzed in cultures with glucose or fructose (100 g/L), with the aim of comparing the fermentation yields under optimal (30 °C) and suboptimal (40 °C) temperature conditions. In general, all analyzed strains at 30 °C showed an exponential growth up to 24 h and entered the stationary phase at 60 h (Fig. 1). Interestingly, Sc3Y8 strain, which exhibited a wide thermotolerance in solid culture, showed the lowest growth under these conditions. At 40 °C, both *Saccharomyces* strains had a lower growth than at 30 °C. Regarding sugar consumption, these strains show different profiles since only the ScFerm completely consumed both sugars at 30 °C (after 30 h), but left 50 and 23 g/L of glucose and fructose, respectively, at 40 °C. The Sc3Y8 strain had a high residual sugar concentration of 17 and 31 g/L of glucose and fructose, respectively, at 30 °C, and overall it was less efficient than the industrial strain at this temperature. At 40 °C the residual sugar concentration was similar to the one of ScFerm. Different fructose uptake at different temperatures has been previously reported by Dumont (*12*), who tested five industrial wine yeast. Fructose and glucose consumption capacity by these industrial strains at increased temperatures was analyzed, with and without nitrogen limitation, and it was observed that glucose was consumed at higher rate at the beginning of the fermentation and fructose at a slower steady rate. Similar results were reported in some mezcal yeasts (*4*), including the Sc3Y8 selected in this study. Additionally, Dumont *et al.* (*12*) observed that at the latter stage of fermentation, the industrial wine yeasts that are considered fructophilic are able to continue uptaking fructose while leaving a higher residual glucose concentration in the medium. The same authors documented that the higher the temperature, the better the fructose consumption by all except one of the tested strains (a low temperature specialist, as stated by the authors), and it is worth noticing that their higher temperature (28 °C) tested is similar to the optimal (30 °C) temperature used in our study. Hence, the results shown in this work concerning an enhanced fructose consumption capacity at high temperatures are indeed one of the most interesting phenotypical characteristics of this mezcal strain. It is clear that more effort must be made to clarify this different kinetic behaviour in relation to fructose in this strain.

Concerning the production of metabolites, at the end of cultivation (60 h), glycerol, ethanol and acetic acid concentrations increased in all selected strains when grown on fructose at 40 °C (Table 2). Sc3Y8 strain showed higher metabolic accumulation at this temperature. These different fermentation profiles as a consequence of the increase in the temperature and the type of used sugar could be due to their different ethanol tolerance and their differences in the hexose transporter efficiencies (*15*, *33*). Interestingly, glycerol was the metabolite with the higher increase (>7 times) at 40 than at 30 °C (Table 2). It has been reported that temperature affects the glycerol accumulation in *S. cerevisiae* strains (*8*) since it acts as an osmoregulator between the cytosol and the environment (*34*). Similar to the accumulation of glycerol, the alcohol and acetic acid levels also increased (2 to 3 times) when these selected strains were grown at 40 °C using fructose as a carbon source, particularly Sc3Y8 strain. In general, the use of fructose at 40 °C increased the metabolite production by the yeasts, similar to what had been previously reported for some industrial *S. cerevisiae* strains (*12*). It is worth noticing that under stressful (temperature, osmotic pressure, *etc*.) conditions, *Saccharomyces* strains tend to overproduce glycerol and acetic acid, and to produce less biomass, as corroborated with the colony count in Table 1, *i.e*. the number of viable cells that grew in glucose or fructose at the two highest temperatures tested for all the strains. This general trend can be observed for both hexoses when comparing metabolite profiles (Table 2) at 30 and 40 °C. These results suggest that the two analyzed strains have a different metabolic response at 40 °C, with the mezcal yeast (Sc3Y8) producing more ethanol at this temperature, probably due to its original niche of isolation, which is a fermentation that is carried out at high fructose concentration and uncontrolled temperature.

**Table 2:** Concentration of metabolites in selected strains after 60 h of fermentation under different temperature conditions

*Saccharomyces* strain	Temperature/°C	Carbon source	*γ*/(g/L)			
Glycerol	Ethanol	Acetic acid	Residual sugar
ScFerm	30	Glucose	1.0±0.2	15.5±1.2	2.2±0.4	0.19±0.1
		Fructose	1.1±0.2	14.3±1	1.8±0.3	5.8±0.3
	40	Glucose	5.4±0.1	26.8±3.4	4.5±1.5	23.4±13.5
		Fructose	8.0±0.1	32.6±1.7	6.4±0.1	43.2±3.1
Sc3Y8	30	Glucose	0.8±0.0	12.5±1.1	1.7±0.4	17.1±3.6
		Fructose	1.5±0.3	15.1±1.5	2.1±0.4	31.4±4.8
	40	Glucose	3.6±2.7	33.3±3	2.1±0.2	34.2±7.8
		Fructose	8.6±0.4	43.6±3.3	7.3±0.5	32.3±0.2

### Flocculation ability

**Fig. 2:**
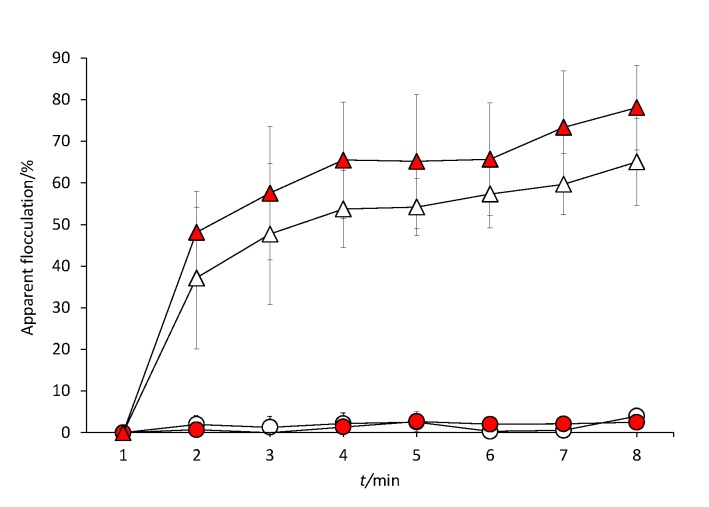
Flocculation phenomenon in the presence of CaCl_2_ in the selected *Saccharomyces cerevisiae* Sc3Y8 (triangles) and ScFerm (circles) strains. White and red symbols indicate the fermentation temperature of 30 and 40 °C, respectively

**Fig. 3:**
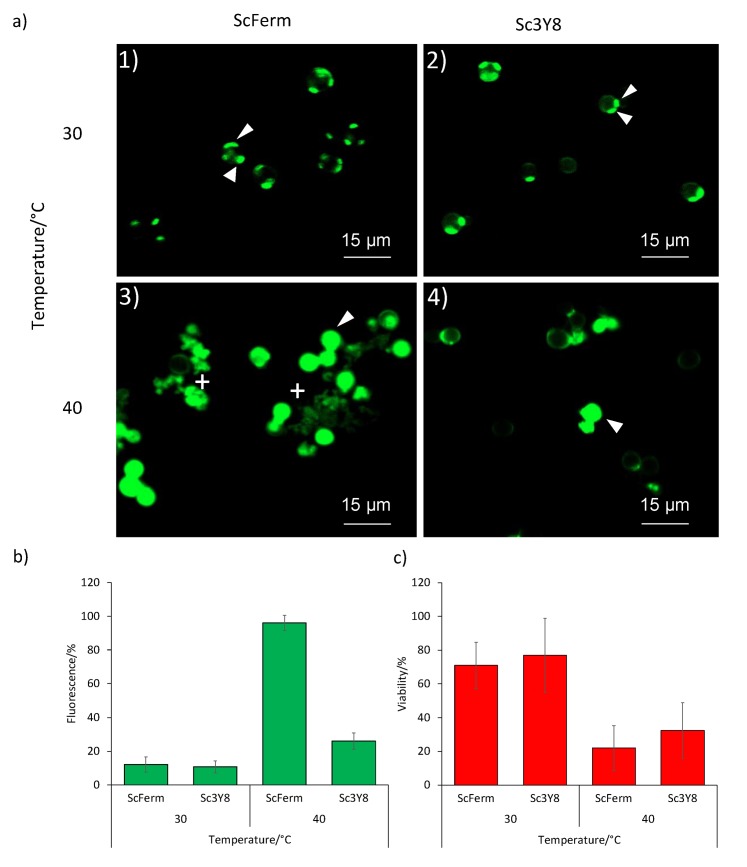
Fluorescence analysis using Solophenyl Flavine: a) Solophenyl Flavine fluorescence in ScFerm (1 and 3) and *Saccharomyces cerevisiae* Sc3Y8 (2 and 4) cells grown at 30 (1 and 2) and at 40 °C (3 and 4) on deionized water without CaCl_2_. Arrows on cells grown at 30 °C show the sites that probably correspond to bud scars, and on cells grown at 40 °C where the signal covers all cell surface area. The crosses show the cell lysis at 40 °C. Relative percentages of: b) fluorescence and c) viability during flocculation assay at different temperatures (30 and 40 °C). Relative fluorescence was calculated on the basis of stained cells compared to the total cell number in 12 microscopic fields. Relative viability was expressed as CFU count in the microdrop assay using the initial inoculum of 5·10^7^ cell/mL. Experiments were done in triplicate

Flocculation has been determined in some yeast species, mainly in *S. cerevisiae*, as a mechanism to overcome stress conditions (*i.e.* alcoholic or nutrient depletion) (*22*, *35*). It was observed only in Sc3Y8 strain (Fig. 2). This phenomenon was CaCl_2_-dependent since it was not observed in the presence of water or buffer without calcium (data not shown). Confocal microscopy analysis of Sc3Y8 strain using the fluorophore Solophenyl Flavine 7GFE 500 (AK Scientific, Inc.) allowed to assess some changes in the cell wall conformation at 40 °C, similarly to the observations made by Soares (*19*), using the fluorophore Calcofluor White M2R in cells that were subject to osmotic stress (1 mol/L NaCl) or a brief heat-shock (52 °C for 5 min). In this study, the temperature stress was maintained during fermentation. The fluorophore Solophenyl Flavine 7GFE 500 has not been previously used in flocculation analysis so it was first tested to determine its incubation time, concentration and viable cell number. The fluorophore did not show adverse effects on the cell viability during or after flocculation assay, so it was suitable for cell observations. It was determined that the best mass per volume ratio of Solophenyl Flavine was 0.4% at an incubation time of 15 min. Observations at 30 °C in the absence of CaCl_2_ evidenced oval structures with a higher fluorescent intensity, likely the scars generated during budding (Fig. 3). In contrast, at 40 °C, the fluorophore recognized the whole cell wall and also allowed to observe cell lysis (Fig 3a). To confirm that the fluorescence detected at 40 °C is a consequence of the cell wall recognition by the Solophenyl Flavine and not their inclusion in the lysed cells, a viability assay was conducted in parallel. Fig. 3b and Fig. 3c compare the fluorescent analysis and the viability determination (plate count). In general, a reduction in the viability at 40 °C was observed although Sc3Y8 strain was slightly more viable than ScFerm (non-flocculent strain used as a control). Regarding fluorescent analysis, similar behaviour was observed between both *S. cerevisiae* strains at 30 °C, but these values increased significantly in the ScFerm strain at 40 °C. Considering the results at 40 °C, the increase in the fluorescence might be a consequence of the reduction in the viability and of the cellular lysis detected in the ScFerm strain, in contrast to the more temperature-tolerant Sc3Y8 strain, which exhibited a lower reduction of their fluorescence even at 40 °C.

Confocal microscopy analysis using the Solophenyl Flavine in the presence of CaCl_2_ showed cell aggregates only in the Sc3Y8 strain. These cell aggregates showed apparent different sizes depending on the temperature conditions (data not shown). Finally, at 40 °C, less lysed cells were observed in the Sc3Y8 than in ScFerm strain, supporting the previous viability assay. These results suggested that the flocculation phenotype in the Sc3Y8 strain may contribute to its thermotolerance due to the changes in the cell wall conformation and integrity, allowing a higher viability. Also, it has been reported that temperature affects the flocculation phenomenon at the level of cellular interactions, probably as a consequence of the flocculin denaturalization (*19*). Flocculation observed in Sc3Y8 cells slightly increased when the temperature was raised from 30 to 40 °C, although it was not statistically significant. Additionally, the floccule size in the Sc3Y8 strain is apparently bigger at 40 than at 30 °C. It has been reported that small changes in the cell wall hydrophobicity can also affect the floccule size (*22*). The differences found in the staining pattern caused by the fluorophore Solophenyl Flavine are not directly correlated with the cell viability (*36*). Nevertheless, it seems to be related to changes in the cell envelope caused by the increase of temperature. Recently, a transcriptomic relationship between the stress response and the flocculation was identified in a wine *S. cerevisiae* (F6789A) strain after deletion of the *FLO5* gene. These genes regulate stress tolerance and are related with cell wall reorganization and adhesion (*26*).

### FLO gene expression at 30 and 40 °C

**Fig. 4:**
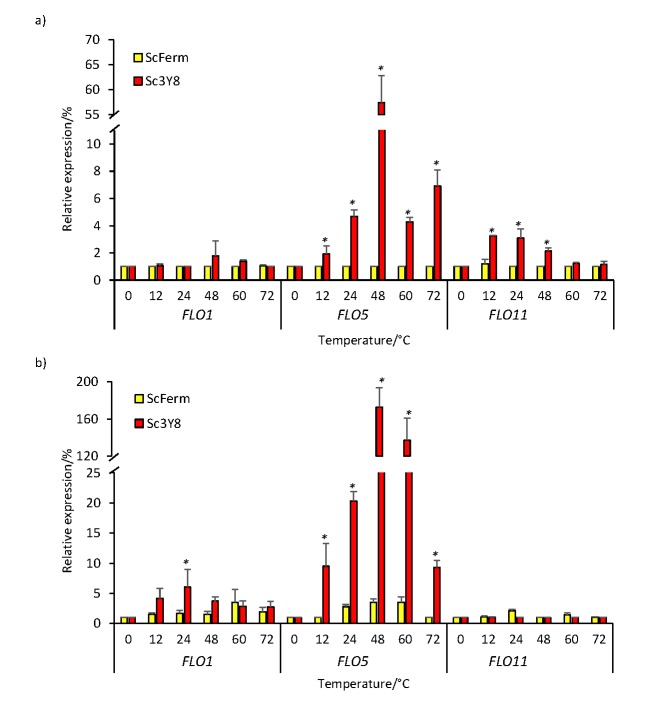
Relative expression of *FLO1*, *FLO5* and *FLO11* genes in ScFerm (yellow) and *Saccharomyces cerevisiae* Sc3Y8 (red) strains grown for 72 h in yeast extract peptone dextrose (YPD) broth (100 g/L) at: a) 30 °C or b) 40 °C, determined by RT-qPCR. Genetic expression was normalized using the constitutive *UBC6* gene. All the analyses were performed with three biological replicates from at least two independent experiments. Error bars represent the standard error (*N*=3). Values with asterisks are significantly different according to the Tukey´s test (p<0.05)

With the aim to better understand the flocculation phenomenon in the Sc3Y8 strain, a *FLO* gene expression analysis was conducted. Genetic regulation, specifically involving lectins, has been related to physiological and epigenetic changes that in turn might be involved in cell wall synthesis (*15*). Gene fragments of the selected *FLO1*, *FLO5* and *FLO11* genes were successfully amplified generating sequence fragments of 68, 193 and 103 bp, respectively (Table S1). *FLO1*, *FLO5* and *FL*O*11* transcript accumulation was analyzed in these *Saccharomyces* strains on YPD broth at 30 and 40 °C. Results showed that *FLO1* gene transcripts were constant at 30 °C in both *Saccharomyces* strains. However, at 40 °C a slight but significant (Tukey's test; p<0.05) induction (6%) was observed after 12 h, particularly in the Sc3Y8 strain. ScFerm also exhibited a reduced induction (3.8%) at 60 h of culture (Fig. 4). A stronger *FLO5* expression in Sc3Y8 strain was induced at 40 (up to 20-fold) than at 30 °C. This transcript induction was statistically different among strains after 12 h, and had a maximal value of 175% at 48 h in the Sc3Y8 strain, which coincides with the beginning of the stationary phase (Fig. 1). The non-flocculent strain (ScFerm) kept constant its *FLO5* gene expression at both temperatures.

Our results for the flocculent Sc3Y8 strain suggest a transcriptional induction, at least of *FLO1* and *FLO5* genes, as a consequence of the temperature increase. Expression of both genes was constant in non-flocculent strain ScFerm, with only a slight increase at 40 °C. *FLO5* gene transcription in Sc3Y8 strain strongly increased at both temperatures, but it was the highest at 40 °C. Di Gianvito *et al.* (*26*) have recently demonstrated the importance of the *FLO5* gene during flocculation and related it to stressful (ethanol at 20%) conditions in different *Saccharomyces* wine strains (*10*). Recently, this gene has been deleted in a *S. cerevisiae* wine strain (F6789A-Δ *flo5*) causing an up-regulation of genes related to assembly, shape and cell adhesion, and genes related to the organization of the cell wall, among others (*26*). The transcription of *FLO1* gene has also been related to flocculation ability in *Saccharomyces* strains, although with a lower transcriptional activation of *FLO5* gene (*10*), in line with our observations. The predictive protein products of *FLO1* and *FLO5* are 96% similar and both are subtelomeric genes but they are not alleles (*37*). The regulated expression of these *FLO1* and *FLO5* genes leads to speciﬁc phenotype intensities, resulting in cell aggregation, cell surface properties, and ﬂocculation (*10*, *23*). Finally, the expression profile of the *FLO11* gene showed that at 30 ° C, there was a slightly higher statistically significant difference in strain Sc3Y8 at 24 and 48 h (increase in expression of 2 and 3.2%, respectively). At 40 °C, there was a constantly low expression of *FLO11* in both strains. This gene has been related to a wide phenotype variety, such as flocculation, the invasive growth and ﬂor formation (*38*), probably depending on the culture conditions. Further studies using other cell aggregation or cell-surface adherence genes as *AMN1* and *FLO8* must be conducted to clarify the relation between the flocculation and the stress response, specifically when these cell properties are involved (*39*).

## CONCLUSIONS

*Saccharomyces cerevisiae* Sc3Y8 mezcal strain increased its metabolite accumulation at 40 °C in the presence of fructose as a sole carbon source, as compared to the control strain. This mezcal strain was also able to flocculate in the presence of calcium ions. Gene expression analysis of the flocculation-related genes *FLO1*, *FLO5* and *FLO11* showed that the Sc3Y8 strain has a different gene expression profile from the non-flocculant commercial wine strain *S. cerevisiae* Fermichamp (ScFerm) industrial strain. Flocculation seems to be associated with morphological cell wall changes that might contribute to its thermotolerant phenotype and to a higher viability, which in turn allows a better fermentation productivity at high temperature. Nonetheless, to corroborate this relation, the construction of *FLO*-specific mutants is required to address their exact role in the thermotolerance observed in the flocculating strains. Indeed, more effort must be made to clarify these technologically important physiological traits.
